# Integrating earthworm movement and life history through dynamic energy budgets

**DOI:** 10.1093/conphys/coac042

**Published:** 2022-06-27

**Authors:** Andre Gergs, Kim Rakel, Dino Bussen, Yvan Capowiez, Gregor Ernst, Vanessa Roeben

**Affiliations:** 1 Bayer AG, Alfred-Nobel-Straße 50, 40789 Monheim am Rhein, Germany; 2 Research Institute for Ecosystem Analysis and Assessment (gaiac), Kackertstrasse 10, 52072 Aachen, Germany; 3 INRAE, UMR EMMAH, 228 Route de l'Aérodrome, 84914 Avignon Cedex 9, France

**Keywords:** temperature, soil moisture, organic matter, reproduction, growth, Aporrectodea caliginosa

## Abstract

Earthworms are considered ecosystem engineers and, as such, they are an integral part of the soil ecosystem. The movement of earthworms is significantly influenced by environmental factors such as temperature and soil properties. As movement may directly be linked to food ingestion, especially of endogeic species like *Aporrectodea caliginosa*, changes in those environmental factors also affect life history traits such as growth and reproduction.

In our laboratory studies, earthworms showed a decrease in burrowing activity with decreasing moisture levels and, to some extent, the organic matter content. The burrowing activity of earthworms was also affected by temperature, for which the casts produced per earthworm was used as a proxy in laboratory experiments. We integrated changes in earthworm movement and life histories in response to temperature, soil organic matter content and the moisture level, as observed in our experiment and reported in the literature, through dynamic energy budget (DEB) modelling. The joint parametrization of a DEB model for *A. caliginosa* based on movement and life history data revealed that food ingestion via movement is an integral part of the earthworms’ energy budgets. Our findings highlight the importance of soil properties to be considered in the model development for earthworms. Furthermore, by understanding and incorporating the effect of environmental factors on the physiology, this mechanistic approach can help assess the impact of environmental changes such as temperature rise or drought.

## Teaser

Earthworms are an important part of the soil ecosystem. For conservation of those ecosystems and management of human activities it is important to understand how earthworms move, grow and reproduce under different environmental conditions. We have developed a simulation model that allows to predict earthworm performance under environmental change.

## Introduction

Earthworms are considered ecosystem engineers and, as such, are an integral part of the soil ecosystem. Their functional importance for organic matter turnover, soil fertility, aeration (Lee, 1985) and water infiltration are closely related to their movement. The movement of earthworms, in turn, depends on their ecological category and preferences. Endogeic earthworms, such as *Aporrectodea caliginosa*, common to arable fields in Europe (Le Couteulx *et al*., 2015; Bart *et al*., 2018; Jouni *et al*., 2018), are predominantly active within the upper soil layer creating branched horizontal burrows (Shipitalo and Le Bayon, 2004; Capowiez *et al*., 2014), while epigeic and anecic earthworms live in the litter layer or are considered deep-burrowing species respectively (Nuutinen and Butt, 2003; Shipitalo and Le Bayon, 2004).

The movement of earthworms is significantly influenced by environmental factors such as temperature and soil properties. Earthworms may be exposed to a wide range of ambient temperatures, depending on their ecotypes. Temperature has been reported to affect burrow rates (Perreault and Whalen, 2006) as well as the distribution of earthworms. While anecic earthworms, such as *Lumbricus terrestris*, avoid cold temperatures by migrating to more favourable soil layers (Edwards and Bohlen, 1996), the endogeic species *A. caliginosa* is considered largely freeze-tolerant (Holmstrup and Overgaard, 2007). The soil organic matter content serves as a proxy for food availability and is one of the main drivers of the burrowing activity in earthworms (Hughes *et al*., 1996; Le Couteulx *et al*., 2015; Frazão *et al*., 2019) as is the soil moisture content (Kretzschmar, 1991; Kretzschmar and Bruchou, 1991); e.g. *A. caliginosa* specimens were observed to cease movement and undergo a diapause at dry conditions (Holmstrup, 2001).

Temperature and soil properties also affect life history traits in earthworms. Somatic growth rates and cocoon production were observed to increase while development times decreased with increasing temperatures within optimal ranges (Reinecke and Kriel, 1981; Holmstrup *et al*., 1991; Butt, 1993; Berry and Jordan, 2001). As for soil properties, earthworm growth and reproduction were shown to depend on the addition of organic matter for instance in the form of manure (Neuhauser *et al*., 1980; Reinecke and Viljoen, 1990; Bart *et al*., 2019) as well as on moisture conditions (Holmstrup *et al*., 1991; Eriksen-Hamel and Whalen, 2006). With respect to conservation physiology, it is important to better understand the impact of environmental change and anthropogenic influence on organism level to potentially be able to predict, e.g. life history traits and species distribution.

Dynamic energy budget (DEB) models allow describing life history traits in relation to changing environmental conditions such as temperature and food availability (e.g. Gergs and Baden, 2021). DEB theory (Kooijman, 1986, 2010; Nisbet *et al*., 2000; Sousa *et al*., 2008; Jusup *et al*., 2017) provides a set of rules that quantify energy metabolism of organisms throughout their life cycle based on the law of conservatism of mass and energy. Energy budgeting in those models allows the prediction of energy assimilation from food and the subsequent allocation of energy to growth, development, reproduction and maintenance of bodily functions. The generality of DEB models allows their application to a wide range of species with a relatively small number of parameters (Sousa *et al*., 2008; Kooijman and Lika, 2014; Kearney *et al*., 2015), including soil organisms such as earthworms (Jager and Klok, 2010; Rakel *et al*., 2020).

In this study we quantitatively link, through DEB modelling, earthworm movement and live histories in relation to environmental factors, i.e. temperature, soil organic matter and moisture content, for the example of the endogeic species *A. caliginosa*. The aim was to explore if the effect of those environmental factors on life history traits can be explained by changes in movement rates of the earthworms. More specifically, our objectives were to (i) conceptualize the integration of soil properties in the DEB model, (ii) perform an estimation of the DEB parameters for *A. caliginosa* and (iii) explore how observations and simulations evidence the link between soil properties, behaviour and energy balance. We illustrate that movement is an integral part of feeding and thus life history performance of earthworms and highlight the importance of considering soil properties in the model development for application in conservation and management. The mechanistic understanding provided by the model and the ability to predict the response of earthworms towards environmental changes and stressors can be a helpful tool for conservation research.

## Material and methods

### DEB model formulations

The current model formulation is based on the standard DEB model (Kooijman, 2010). The earthworms are represented by four primary state variables (Table 1): (i) the structural length, L; (ii) the reserve energy, E; (iii) the maturity, E_H_; and (iv) the reproduction buffer, E_R_. Model equations (Table 2) link environmental input variables, i.e. temperature, moisture and organic matter content, to apical endpoints such as weights or burrow rates (Table 1). Model parameters are described in Table 3.

**Table 1:** Inputs, intermediates, state variables and model outputs

Symbol	Description	Unit
*B*	Burrow rate	cm d^-1^
*D*	Fraction of diapausing worms	-
*E*	Reserve energy	J
*E_H_*	Maturity level	J
*E_R_*	Reproduction buffer	J
*f_OM_*	Scaled functional response related to organic matter content	-
*f_M_*	Scaled functional response related to soil moisture	-
*F_t_*	Temperature correction factor	-
*J_x_*	Ingestion rate	g d^-1^
*L*	Volumetric structural length	cm
*L_w_*	Measured body length	cm
*M*	Moisture content	%
*OM*	Organic matter content	%
*pOM*	Organic matter ingestion rate	J d^-1^
*pA*	Assimilation flux	J d^-1^
*pC*	Mobilization flux	J d^-1^
*pJ*	Energy invested in maturity	J d^-1^
*p_X_*	Organic matter assimilation	J d^-1^
*r*	Specific growth rate	d^-1^
*T*	Ambient temperature	°C
*W_d_*	Dry weight	g
*W_w_*	Fresh weight	g
*X*	Food density in the environment	J cm^-3^

**Table 2:** Model equations (Eq.) for energy acquisition and use, movement and temperature correction

Equation	No.
Movement, feeding and assimilation	
*p_OM_ = p_am_ L*^2^*X* (*x_K_ + X*)^−1^ with *x_K_ = p_am_ F_m_*^−1^ and *X = μ_OM_ OM*	Eq. 1
*p_X_ = p_OM_ κ_x_^−1^*	Eq. 2
*Jx = μ_OM_ p_X_*	Eq. 3
*f_OM_ = p_X_ p_Am_*^−1^ *L*^−2^	Eq. 4
*B = k_b_ L^2^ max(0, M - m_0_)*	Eq. 5
*D = exp(− b_w_ max(0, M - m_0_))*	Eq. 6
*f_M_ = B b_max_*^−1^ *L^−2^*	Eq. 7
Reserve dynamics and energy fluxes	
d*E/*d*t = p_A_ − p_C_*	Eq. 8
*p_A_ = f_OM_ f_M_ p_am_ L*^2^	Eq. 9
p_C_ = E * (v/L − r)	Eq. 10
*p_J_ = H κ_J_*	Eq. 11
Growth	
*r = (E v L^−4^ - p_M_ κ^−1^) (E L^−3^ + E_G_ κ^−1^) ^-1^*	Eq. 12
d*L/*d*t = r/*3 *L*	Eq. 13
Maturation and reproduction	
*dE_H_/dt = max(0, (1 − κ) p_C_ − p_J_)* for *H <*${EH}_p$	Eq. 14
*dE_R_/dt = κ_R_ max(0, (1 − κ) p_C_ − p_J)_* for *H ≥*${EH}_p$	Eq. 15
*Eggs = E_R_*${U_E^0}^{-1}$ {*p_Am_*}^−1^	Eq. 16
Length conversions	
*L_w_ = L δ_M_^−1^*	Eq. 17
*W_d_ = L^3^ δ_V_ (1+ w f)*	Eq. 18
*W_w_ = L^3^ (1+ w f + w_V_ (1 − f))*	Eq. 19
Temperature	
*s_A_ = exp(T_A_ T_ref_^−1^ - T_A_ (T + 273.15) ^-1^)*	Eq. 20
*S_rH_ = (1 + exp(T_AH_ T_H_^-1^ − T_AH_ T_ref_^−1^)) (1 + exp(T_AH_ T_H_^-1^ − T_AH_ (T + 273.15) ^-1^)) ^-1^*	Eq. 21
*F_t_ = s_A_ ((T + 273.15 ≥ T_ref_) s_rH_ + (T + 273.15 < T_ref_))*	Eq. 22

**Table 3:** Model parameters, descriptions and values for a reference temperature (*T_ref_*) of 20°C

Parameter	Description	Unit	Value
b_w_	Distribution width for diapause	%^−1^	0.78
EH_b_	Maturity at birth	J	16.79
EH_p_	Maturity at puberty	J	1131
E_G_	Specific costs for structure	J cm ^−3^	4194
f	Scaled functional response for zero variate data	-	1
F_m_	Maximum specific searching rate	g d^−1^ cm^−2^	85.78
k_J_	Maturity maintenance rate coefficient	d^−1^	0.0028
m_0_	Moisture threshold for movement	%	8.69
*b_max_*	Maximum moisture dependent specific movement rate	cm(soil) d^−1^%^−1^ cm^−2^	87.98
*p_am_*	Surface area specific maximum assimilation flux	J d^−1^ cm^−2^	1673.9
p_M_	Volume specific somatic maintenance costs	J d^−1^ cm^−3^	1560
*k_b_*	Moisture-dependent specific movement rate	cm(soil) d^−1^%^−1^ cm^−2^	6.205
T_A_	Arrhenius temperature	K	7976
T_AH_	Arrhenius temperature for upper boundary	K	28 750
T_H_	Upper temperature boundary	K	293.20
T_ref_	Reference temperature	K	293.10
${U}_E^0$	Cost for an egg	cm^2^ d	0.078
v	Energy conductance	cm d^−1^	0.017
w	Contribution of reserve to body fresh weight	g cm^−3^	27.24
w_V_	Contribution of water replacement to body fresh weight	g cm^−3^	6.72
*δ*_M_	Shape coefficient	-	0.065
*δ*_V_	Specific density of structure	g cm^−3^	0.27
*κ*	Allocation fraction to soma	-	0.40
*κ*_R_	Deproduction efficiency	-	0.48
*κ*_X_	Digestion efficiency of food to reserve	-	0.26*
*μ_OM_*	Organic matter - energy coupler	J g^−1^%^−1^	186.20**

a ^*^ From Whalen and Parmelee, 1999.

b ^**^ From Lavelle and Spain, 2001.

We here extended the standard DEB model by explicitly simulating the movement of earthworms through the soil and the associated ingestion food (Eq. 1–7) to calculate scaled functional responses related to soil moisture and organic matter content: modelled food ingestion follows a functional response of organic matter content based on the squared structural length (Eq. 1–3). For the calculation of the functional response, we employ of a factor μOM to convert the soil organic matter content in terms of mass into the unit of energy. The scaled functional response is subsequently derived by dividing the actual assimilation by the maximum assimilation rate (Eq. 4). Cast production is simulated based on the assumption that cast weight equals the soil ingestion and that the weight contribution of assimilated energy is negligible (Eq. 3). Furthermore, we assume that the burrow rate of the earthworms follows a threshold function of moisture content and the squared structural length of the worms (Eq. 5). In case moisture content hits the threshold for burrowing, the chance for a worm in the tested population to be diapausing (or rather entering a period of quiescence) is decreasing with increasing moisture content (Eq. 6). A scaled functional response for moisture is derived by dividing the actual burrow rate by a maximum surface area specific burrow rate (Eq. 7).

Reserve utilization, growth, maturation and reproduction as well as length conversions and temperature correction follow the standard DEB model (Eq. 8–22): reserve dynamics and energy fluxes are given by Eq. 8–11. Note that we multiplied the scaled functional responses for organic matter and moisture to derive the assimilation flux p_A_ (Eq. 9). The equations for growth maturation and reproduction (Eq. 12–16) follow the standard DEB model. Physical (measured) length and dry weight of earthworms is derived from structural length using equations Eq. 17 and Eq. 18, respectively. For the calculation of fresh weights, we assumed that under intermitted situation of weight losses the reduced reserve density is partly replaced by water (Eq. 19); for a discussion see Rakel *et al*. (2020). In the model, all rate constants were corrected by ambient temperature (Eq. 20–22). Rates increase with temperature in an exponential manner until an upper temperature boundary is reached and the temperature response is inverted. The model was implemented in Matlab using DEBtool (available online at https://github.com/add-my-pet/DEBtool_M). Model parametrization was done using the Nelder–Mead simplex algorithm as implemented in DEBtool. For model evaluation, the mean residual error and the symmetric mean squared error were calculated as implemented in DEBtool.

### Experimental data

Model parametrization is largely done based on data available from the literature (Holmstrup *et al*., 1991; Holmstrup, 2001; Bart *et al*., 2019); raw data were kindly provided by the authors. The cocoon development time (age at birth in a DEB context) were quantified by Holmstrup *et al*. (1991) at a range of 5–20°C (for details on the experimental setup, see Holmstrup *et al*. (1991)). Data on life history parameters in relation to soil moisture originated from the study of Holmstrup (2001). In this study, the author assessed the effect of different soil moisture levels on the percentage of diapausing worms [%], the juvenile weight [g], the adult weight [g] and the reproductive rate [#/week] in two different soil types (for more details on the material and methods of this study, see Holmstrup, 2001). The study of Bart *et al*. (2019) provided data on the growth (measured as weight) and reproduction of *A. caliginosa* at different food qualities and quantities. Experiments were conducted in meadow sampled Luvisol soil from Versailles with hand sorted individuals from an agricultural field at the research facility (for details on the study site and soil conditions, see Bart *et al*., 2019). The here presented approach uses the growth and reproduction data of worms fed with horse dung at either 1 or 3 g/individual/14 days. Worms were kept either individually or grouped after puberty. The grouped earthworms produced cocoons and their output was also recorded.

Three additional experiments with *A. caliginosa* were conducted in the here presented study to determine burrowing activity in relation to environmental factors: cast production was quantified as function of temperate and burrow rates were measured as function of soil organic matter and moisture contents. Prior to experiments, *A. caliginosa* specimens were collected in the field as described in Capowiez *et al*. (2021) and acclimatized to laboratory conditions. Experiments were carried out using field sampled soil from an abandoned orchard near Avignon (SE of France). The soil was identified as a sandy clay loam with 29.2% clay, 52.4% silt, 18.4% sand, pH = 8.3, 1.54% organic carbon content, 0.18% total nitrogen and 24.7 g/100 g water holding capacity (see also Supplementary Material Fig. S1; Capowiez *et al*. 2021).

For the quantification of cast production, *A. caliginosa* specimen (*n* = 8, 0.42 ± 0.07 g fresh weight) were placed in petri dishes (10 × 3 cm) filled with soil, kept at seven different ambient temperatures in the range of 2–32°C, and the fresh weight of casts was quantified at test termination (for further details, see Capowiez *et al*., 2010). Burrow rates in *A. caliginosa* in relation to soil organic matter (*n* = 4–5) and moisture contents (*n* = 8) were quantified in 2D terraria, also referred to as Evans boxes (see e.g. Butt and Grigoropoulou, 2010). The terraria (42 × 29 × 0.5 cm) were filled with field sampled soil, but the soil organic matter and soil moisture content was varied. Burrowing activities of individual worms were quantified as described in Grigoropoulou *et al*. (2009).

**Figure 1:**
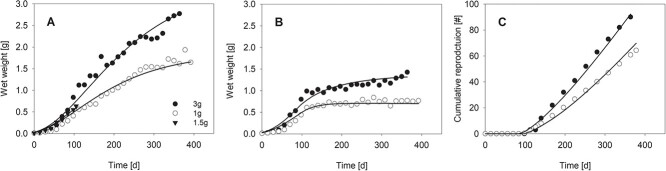
Growth of (**A**) individually kept worms and (**B**) worms that were grouped after puberty, as well as (**C**) reproduction of *A. caliginosa* pairs. Worms were fed with horse dung at different rates (1–3 g/individual/14 days). Dots represent data from Bart *et al*., 2019; lines are the model fits. A reduced value for scalded functional response after puberty, compared with individually kept worms, was assumed for the simulations shown in (B) and (C).

To determine the effect of quantity and location of organic matter, four treatments were tested in the experiment to mimic more realistic conditions: OM-rich soil, OM-poor soil, tilled soil and no-till soil. For the OM-rich and OM-poor treatments, the soil organic matter content was amended to 4% and 2% with an even distribution in the 2D terraria. For the tilled soil treatment, the soil in the bottom half of the terrarium was amended to 2% OM, while the upper half contained 4% OM. The no-till soil treatment included 8% OM in the top 5 cm of the 2D terraria, while the rest of the soil contained 4% OM. Burrowing activities were described as total length burrowed, length burrowed in top half and length burrowed in the top 5 cm. The experiments were carried out at 20°C and soil water content of 20%, i.e. under optimal conditions. The burrow rates in relation to soil moisture were determined in a choice experiment. The terraria were divided into two vertical halves with two different soil water contents. The following water contents were tested: 10%, 15%, 20% and 25% (relating to 40%, 60%, 80% and 100% of the water-holding capacity of the soil). In total, five treatments were experimentally tested: 15% vs. 20%, 25% vs. 20%, 25% vs. 15%, 15% vs. 10% and 20% vs. 10%. Burrowing activities were described as total length burrowed per day and total length burrowed. The experiments were carried out at 20°C and organic matter content of 4%. To compare the experimental results with the data from the literature, the units of the soil moisture content were unified to water content [%]. Details on the calculations can be found in the Supplementary Material.

## Results

**Figure 2:**
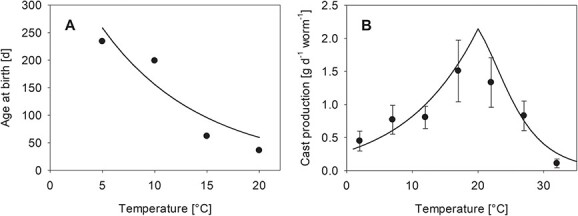
Temperature responses in terms of (**A**) cocoon development time (data from Holmstrup *et al*., 1991) and (**B**) cast production (this study, mean and standard deviation of *n* = 8). Dots and lines represent data and model fits, respectively. For the calculation of the cocoon development time (age at birth) we estimated a delay for the start of development of t_0_ = 40.2 days.

**Figure 3:**
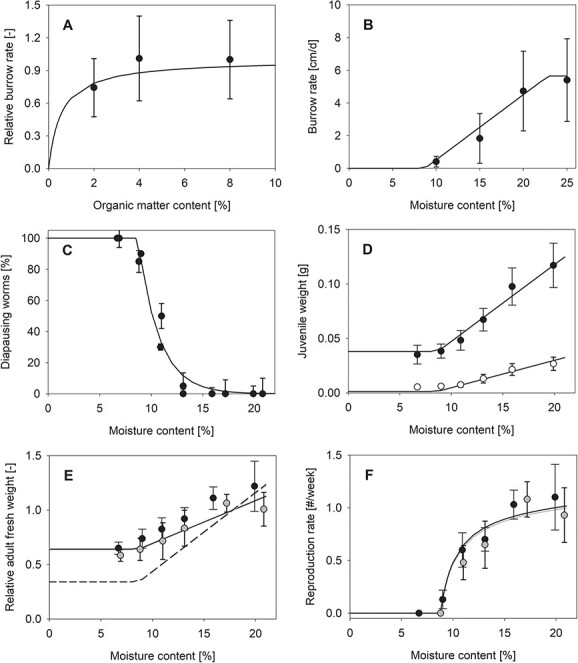
Movement and life history traits as function of soil properties. Dots represent data, and lines are the model fits for (**A**) relative burrow rate as function of organic matter content (means and standard deviation) – relative burrow rate allowed for a functional response scaled from 0 to 1; (**B**) burrow rate of individual worms in response to soil moisture content (means and standard deviation); (**C**) fraction of diapausing worms in relation moisture content; (**D**) juvenile fresh weight (black dots) and dry weight (open dots) for different moisture conditions; (**E**) change in weight relative to initial without (dashed line) and with (solid line) consideration of the gut content for two different soil organic matter contents (grey dots: 3%, black dots: 4%); data in (C)–(F) from Holmstrup (2001).

The DEB model for *A. caliginosa* was simultaneously calibrated to life history data and data related to movement activity. Both data types were quantified for a wide range of environmental conditions regarding food availability, temperature and moisture content (Figs 1–3). The resulting parameter values are listed in Table 3; the mean residual error (MRE) and the symmetric mean squared error (SMSE), both serving as goodness of fit measures, were MRE = 0.111 and SMSE = 0.103. Growth and reproduction trajectories for different food levels (Fig. 1) and zero-variate data (Table 4) were fitted well by the model. We assumed a scaled functional response of *f* = 1 for zero-variate data (Table 4) and the 1.5 g/individual/14 days food addition treatment in the experiments from Bart *et al*. (2019). As the individually kept worms grew to exceptional high weights in the experiments by Bart *et al*. (2019) (see discussion below), we allowed the scaled functional response to reach values larger than one. For individual worms, a value of *f* = 1.1 was estimated for the 3 g/individual/14 days food addition treatment, while it was *f* = 0.85 for the 1 g/individual/14 days treatment (Fig. 1A). In this experiment, another set of worms got paired for reproduction after puberty. We assumed lower scaled functional responses from this point in time to capture the reduced growth compared with individually kept worms. The estimates for scaled functional responses after puberty were *f* = 0.87 and *f* = 0.73 for the 3 g/individual/14 days treatment and 1 g/individual/14 days treatment, respectively (Fig. 1B). The reproduction of the paired worms and different food treatments in the experiment was well described by the model under these assumptions (Fig. 1C).

The cocoon development time (age at birth in a DEB context) as quantified by Holmstrup *et al*. (1991) decreased with increasing temperature in the range of 5–20°C (Fig. 2A). The same Arrhenius temperature (Table 3) as for age at birth (Fig. 2A) described the increase in cast production, a proxy for movement activity, well (Fig. 2B). However, cast production decreased beyond an estimated temperature boundary of 20.05°C (293.2 K; Table 3), which was well described by the three-parameter temperature Arrhenius model (Eq. 20–22; Fig. 2B).

Burrow rates of *A. caliginosa* specimen increased with both organic matter content and soil moisture. Organic matter content here served as a proxy for energy availability and thus a scaled functional response model was used to fit the data. The model describes that data well (Fig. 3A) but note that there were only small differences between the relative burrow rates quantified for the different moisture conditions. There is, thus, some uncertainty regarding the value for the maximum specific searching rate (Table 3) that is used to derive the half saturation coefficient of the functional response (Eq. 1). In contrast to the functional response, the moisture-related burrow rate of *A. caliginosa* specimen increased with moisture content beyond a threshold until a maximum burrow rate is reached (Fig. 3B). The same moisture threshold for movement of m_0_ = 8.67% (Table 3) was used to describe the percentage of diapausing worms for different moisture conditions (Fig. 3C) as measured by Holmstrup (2001). Note that the moisture-dependent movement rates (Table 3; as visible in Fig. 3B) were simultaneously estimated from the burrowing data as well as the growth and reproduction data (Fig. 3B, D–F).

**Table 4:** Zero-variate data used for model parameterization; for zero-variate data a scaled functional response of *f* = 1 has been assumed

Data type	Measured value	Fitted value	Unit	Reference
Time since birth at puberty	91	92.9	d	Bart *et al*., 2018
Length at birth	1.2	1.5	cm	Sims and Gerard, 1999
Ultimate total length	8.5	6.7	cm	Bart *et al*., 2018
Fresh weight at birth	0.03	0.03	g	Boström, 1987
Fresh weight at puberty	0.5	0.5	g	Lofs-Holmin, 1983
Ultimate fresh weight	2	2.3	g	Lofs-Holmin, 1983

The weights of juvenile worms after 28-day exposure to different moisture conditions is well described by the DEB model (Fig. 3D). The comparison of the model output with the initial fresh weight of 0.048 g revealed that body weight was reduced at low moisture and somatic growth occurred at higher moisture levels. In this experiment, worms could empty their guts prior to measurements. To adequately capture the magnitude in loss of fresh weight, we needed to assume that the reserve volume got partly replaced by water (Eq. 19). The same applies to simulation of adult fresh weights (Fig. 3E). In the original experiment (Holmstrup, 2001) with two different soils (3% and 4% organic matter content), adult worms could grow and reproduce for a 14-day period before being transferred to different intermittent moisture conditions for another 14 days. The change in fresh weight and the reproduction rate under these conditions could be well described by the model (Fig. 3E, F), under the following assumptions. As in the experiment total fresh weight (including gut content) was determined and the net worm weight was unknown, we estimated the initial structural length (L_0_ = 0.24 cm) based on the growth and reproduction data and calculated the change in fresh weight relative to initial. We additionally estimated the contribution of the gut content the total fresh weight to be 0.3 g (Fig. 3E, solid line); without consideration of the gut content, the relative change in modelled weight in response to the moisture content would be larger (Fig. 3E, dashed line).

## Discussion

Earthworm movement and life history traits in response to different environmental conditions in terms of temperature, soil organic matter content and soil moisture were well integrated by our DEB model approach.

The weight gain of individually cultured worms in the experiments by Bart *et al*. (2019) exceeded the maximum individual worm weight used in the zero-variate data (Table 1; Lofs-Holmin, 1983) and other studies (e.g. Ernst *et al*., 2022). We thus allowed the scaled functional response for this experiment to exceed a maximum value of 1. In contrast, paired individuals did not reach the maximum weight of 2 g (Table 1; Lofs-Holmin, 1983) also in the high food level in the experiment by Bart *et al*. (2019). To capture this discrepancy, we assumed that the scaled functional response after pairing is lower compared with the individual worms. Similarly, Neuhauser *et al*. (1980) found that maximum body weights in *Eisenia fetida* were higher for individually cultured worms compared to grouped worms. Based on this observation, Johnston *et al*. (2014) argued that reproduction takes priority over growth and that the kappa-rule of the DEB model is not applicable to earthworms. The kappa-rule is a central assumption of the standard DEB model saying that fixed fractions of the reserve are allocated to somatic maintenance and structure on the one hand and to maturity maintenance, maturation and reproduction on the other hand. Explicitly simulating experimental feeding regimes, feeding rates of individual worms and subsequent resource depletion and renewal in experimental setups, however, revealed that the reduced weight gain in grouped worms might be the result of competition for food (Rakel *et al*., 2020), which supports our assumption of the reduced scaled functional responses after paring at puberty (Fig. 1).

In their experiment, Bart *et al*. (2019) successively pooled worms after puberty in groups of increasing numbers of individuals. The amount of food added to the system was adapted accordingly: in each treatment a fixed amount of food per individual was added every 14 days. This setup accounted for the increasing number of worms per system but did not consider that larger worms require more food and could be food limited later in the experiment. Our modelling results suggest that, based on the surface area specific maximum assimilation flux *p_Am_* and the calculated values for length at puberty *L_p_* and length at birth *L_b_* at *f* = 1, the individual energy requirement is more than seven times higher at puberty compared with birth. We thus assumed that individual food intake in the experiments by Bart *et al*. (2019) was reduced after pairing and, as an approximation, estimated lower values for the scaled functional response compared with the juvenile period, which described the data overall well (Fig. 1). An alternative explanation for exceptionally large weights in the study by Bart *et al*. (2019), which could also explain the weight difference to paired worms, would be that non-reproductive individuals accumulate energy in their reproduction buffer considerably contributing to weight if not emptied in the absence of reproduction, which needs further investigation. This possibility is not considered in our model, as the contribution of the reproduction buffer to weight is assumed to be negligibly small in reproducing adults and thus ignored (Eqs. 18 and 19). Considering the contribution of the reproduction buffer to weight would slightly affect the DEB parametrization, but not the integration of the DEB approach and the earthworm movement.

Cast production, or more generally egestion, has previously been used to quantify, e.g. burrowing behaviour in earthworms (Capowiez *et al*., 2010) or feeding rates in sediment dwellers (Forbes and Lopez, 1987). Cast production has been shown to increase with temperature in earthworms (Whalen *et al*., 2004) as did developmental rates (Holmstrup *et al*., 1991). More generally, different physiological rates within one species, e.g. ingestion rate, growth rate, reproduction rate and ageing rate, follow the same Arrhenius temperature, as illustrated for water flea (Kooijman, 2010) and supported by our results for cocoon development and cast production in *A. caliginosa* (Fig. 2).

Endogeic earthworm species such as *A. caliginosa* feed on organic matter present within the soil. The distribution of organic matter in the soil column, e.g. tilled vs. not tilled soil, affects their movement behaviour (Capowiez *et al*., 2021), which was also observed in our study. However, for the parametrization of the model, the focus was on the relative burrowing rate of the earthworms and, thus, the distribution of the individuals was not evaluated. The relative burrowing rate was hardly affected by the difference in organic matter content in the range of 2–8%. The functional response was used to fit the data (Fig. 3A) out of theoretical considerations as ingestion generally follows a decelerating function of resource density, but, as pointed out above, the data had little information on burrowing rates at low organic matter contents. However, low organic matter contents are largely avoided by *A. caliginosa* specimen (Capowiez *et al*., 2021) and might thus be of low ecological relevance for the species.

The soil moisture was a major driver for the burrowing behaviour of *A. caliginosa* specimen in our experiments as well as in other studies (e.g. Holmstrup, 2001). Interestingly, burrowing rates (Fig. 3B), the fraction of diapausing worms (Fig. 3C) and growth and reproduction rates (Fig. 3D–F) could be well described by the same threshold for the moisture content. However, Holmstrup (2001) argued that small changes in the soil water potential are the cause for changes in those rates rather than the moisture content itself. He observed that that nearly all earthworms halted movement at a soil water potential of −40 kPa. Based on the water retention curves, this translates to a water content of 22% for the sandy clay loam used in the laboratory studies of the here presented paper (Supplementary Material Fig. S2). However, no decrease in burrowing activity was documented at this level.

In general, water availability in soil plays an important role for earthworms and their activity (Kretzschmar and Bruchou, 1991). Their metabolism requires water, which is supplied through, e.g, the ingestion of wet soil (Kretzschmar and Bruchou, 1991), and the soil matric potential is thus a more meaningful expression of biologically available water for earthworms (Eriksen-Hamel and Whalen, 2006; Perreault and Whalen, 2006). However, our modelling approach revealed that the same threshold value for moisture contents described various responses in *A. caliginosa* well, which would not be possible when using the soil water potential as an environmental forcing. At the threshold level, the soil water potential differs from the moisture content by >2 orders of magnitude when comparing the soils used in Holmstrup (2001) and our study (Supplementary Material Fig. S2). For details see the supplementary information. Note however that, while the water retention curves are a powerful tool to include study data that did not measure the soil water potential, they carry some uncertainty. The moisture characteristic curves usually describe the desorption curve and cannot account for hysteresis between drying and rehydration curves (Holmstrup, 2001). Supplementary Material Figure S2 reveals thus some deviation between measured and calculated curves for the two soils studied by Holmstrup (2001). Nevertheless, the soil water potential is rarely measured in laboratory or in field studies (Reinecke and Venter, 1987; Lowe and Butt, 2005). Therefore, and from a modelling perspective, it is more practical to use the water content [%] instead of the soil water potential. The here presented model was able to describe the decrease in burrowing activity of *A. caliginosa* for all three soil types well based on the water content [%], resulting in a threshold of 8.67%.

In both experiment and DEB model, burrowing rates are increasing with moisture content and so do growth and reproduction rates increase as consequence of the increased ingestion rates (as revealed by the model analysis). Movement and biological production rates are limited by the worm’s capacities for burrowing as well as for energy assimilation, which intuitively makes sense and they are likely the reason for the decelerating reproduction rates with increasing moisture contents in the study by Holmstrup (2001; Fig. 3F). If moisture conditions become unfavourable and food ingestion decreases compared with previous conditions, the worms may drop in weight due to the depletion of their reserve, which contributes to the total body weight. We assumed that the freed reserve volume is partially replaced by water as discussed by Rakel *et al*. (2020). Evidence for this water-replacement hypothesis comes from the model result for fresh weights and dry weights of juvenile worms (Fig. 3D): The modelled weight loss at low moisture content would have been larger without this replacement assumption as also illustrated by Rakel *et al*. (2020); however, the additional weight was not needed to describe the effect of varying moisture contents on the juvenile dry weight (where water content got excluded from weight measurements). Note, however, that we, for reasons of simplicity and in the absence of more detailed data, assumed that the water replacement is independent of the soil moisture content, which is likely not realistic under dry conditions and may need further investigation.

In conclusion, we have illustrated that, in endogeic earthworms, movement is an integral part of feeding and thus live history traits, which in turn depends on environmental conditions such as temperature, organic matter content and soil moisture. Moreover, we illustrated how movement behaviours in relation to various environmental factors may be integrated in DEB models for earthworms and, due to their generic nature, other soil species. The DEB model may be applied as a module in the chemical risk assessment (e.g. Forbes *et al*., 2021; Roeben *et al*., 2020) as well as in a conservation and environmental change context. In particular, our approach contributes to understanding important aspects of conservation physiology as highlighted by Cooke *et al*. (2013), including understanding influences, stress responses and tolerances to variations in habitat quality and anthropogenic environmental changes, including climate change.

## Data Availability

Data and model code will be made available through the add-my-pet collection at: https://www.bio.vu.nl/thb/deb/deblab/add_my_pet/species_list.html.

## Supplementary Material

supp_coac042
