# Supplementary material for: Integrating earthworm movement and life history through dynamic energy budgets
Source: Conserv Physiol. 2022 Jun 27;10(1):coac042. doi: 10.1093/conphys/coac042 (PMC9235907; doi:10.1093/conphys/coac042)
Supplement: supp_coac042 [file supp_coac042.zip › Supplementary Material.pdf]

Supplementary Material to

Integrating earthworm movement and life history through dynamic energy budgets

Andre Gergs[1], Kim Rakel[2], Dino Bussen[2], Yvan Capowiez[3], Gregor Ernst[1], Vanessa Roeben[1]

[1] Bayer AG, Alfred-Nobel-Straße 50, 40789 Monheim am Rhein, Germany

[2] Research Institute for Ecosystem Analysis and Assessment (gaiac), Kackertstrasse 10, 52072 Aachen, Germany

[3] INRAE, UMR EMMAH, 228 route de l'Aérodrome, 84914 Avignon Cedex 9, France

In order to calculate the soil water potential for laboratory studies where the percentage of wet soil mass had been quantified, we applied a method similar to the one proposed by Perreault and Whalen (2006).

The soil moisture was converted to soil water potential using parameters from the ROSETTA software program (Schaap et al. 1998) based on the USDA soil texture triangle (Fig. S1). A lookup table provided the class-average values of the seven hydraulic parameters for the twelve USDA texture classes. Based on these parameters the van Genuchten function was applied. Following assumptions were made: as suction pressure head is always negative, h2 is used for calculations. For theta < theta_r a boundary was set close to negative infinity and for theta > theta_s a boundary was set close to infinity.

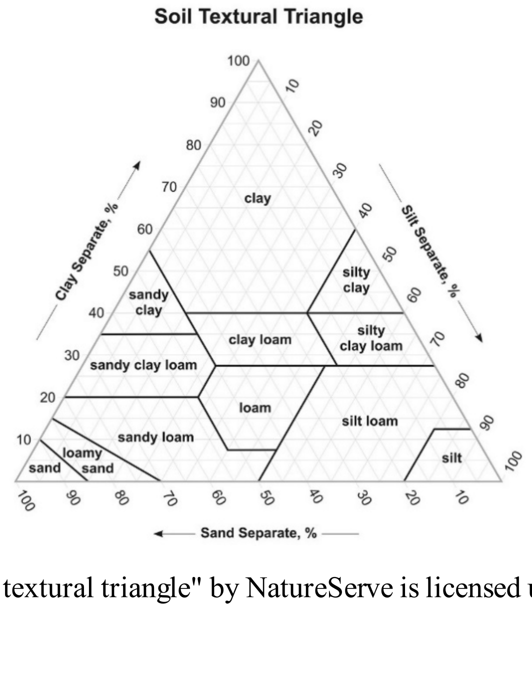

Figure S1. "Soil textural triangle" by NatureServe is licensed under CC BY 2.0

Based on these assumptions, soil moisture levels were calculated to soil water potentials.

$$\theta_h = \theta_r + \frac{(\theta_s - \theta_r)}{[1 + (\alpha h)^n]^m} \quad \text{with}$$

$$m = 1 - 1/n$$

Where θ_h is the water retention curve, θ_r is the residual water content, θ_s the saturated water content, α and n are van Genuchten (1980) parameters, and h is the pressure head or cm of water. For recalculating the relative soil moisture to the soil water potential, the equation was rearranged to

$$h = \frac{\sqrt[n]{\sqrt[m]{\frac{\theta_s - \theta_r}{\theta - \theta_r}} - 1}}{\alpha} * f$$

where f is a conversion factor of 0.0980665 to calculate from cmH_20 to kPa[1].

Based on the soil composition, the soil texture classes of the experimental soil (sandy clay loam) and the soils used by Holmstrup (2001) (loamy sand; sandy loam) were determined and the water retention curves calculated (Fig. S2). The conversion was verified by comparing the retention curves of the original formula (from soil water potential to water content) and the conversed formula (from water content to soil water potential).

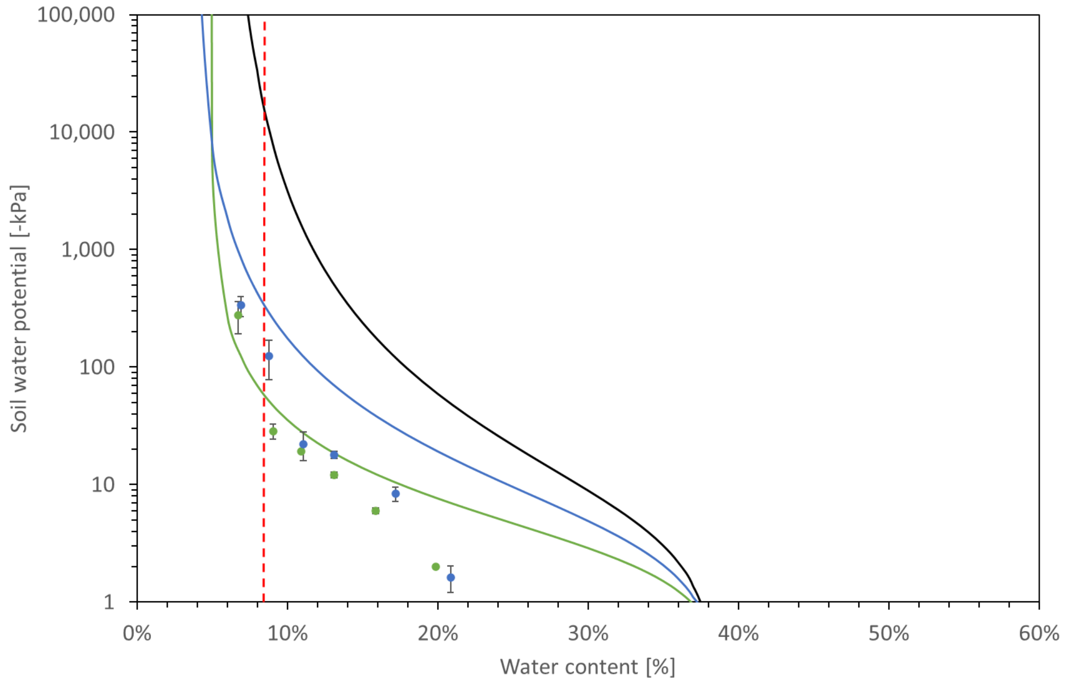

Figure S2: Water retention curves for different soil types. Solid lines are calculated values, dots indicate the measured data of Holmstrup (2001) with confidence intervals. Curves and measurements of soils used by Holmstrup (2001) are represented in green (loamy sand) and blue (sandy loam). Soil used in the experiments described in Material and Methods is represented in black (sandy clay loam). The red dashed line marks the burrowing threshold determined by our model of 8.67% water content.

[1] Furey, Edward "Pressure Conversion Calculator" at https://www.calculatorsoup.com/calculators/conversions/pressure.php from CalculatorSoup, https://www.calculatorsoup.com - Online Calculators

References:

Perreault JM, Whalen JK. 2006. Earthworm burrowing in laboratory microcosms as influenced by soil temperature and moisture. Pedobiologia. 50(5):397–403.

Holmstrup M (2001) Sensitivity of life history parameters in the earthworm Aporrectodea caliginosa to small changes in soil water potential. *Soil Biol Biochem* 33(9):1217–1223.

Schaap MG, Leij FJ, van Genuchten MTh. 1998. Neural Network Analysis for Hierarchical Prediction of Soil Hydraulic Properties. Soil Science Society of America Journal. 62(4):847–855.

van Genuchten MTh. 1980. A Closed-form Equation for Predicting the Hydraulic Conductivity of Unsaturated Soils. Soil Science Society of America Journal. 44(5):892–898.
